# Complete Genome of *Bacillus megaterium* Podophage Pony

**DOI:** 10.1128/genomeA.00860-13

**Published:** 2013-12-05

**Authors:** Brontee E. Khatemi, Christopher C. Chung On, Karthik R. Chamakura, Gabriel F. Kuty Everett

**Affiliations:** Center for Phage Technology, Texas A&M University, College Station, Texas, USA

## Abstract

*Bacillus megaterium* podophage Pony was isolated from a soil sample collected in College Station, TX. Here, we report the sequencing and annotation of the 39,844-bp genome of phage Pony and describe the major features identified.

## GENOME ANNOUNCEMENT

*Bacillus megaterium* is a widely employed organism in the study of microbiology and genetic processes. Phages infecting *B. megaterium* can be used as genetic tools to expand the array of uses of *B. megaterium* in research and industry alike (1). Pony is a podophage, as determined by transmission electron microscopy performed at the Microscopy and Imaging Center at Texas A&M University. Pony has a broad host range of *B. megaterium* strains, including Km Sp^-^, DSM 337, MC 2, QM B1551, PV361, WH320, and WSH-002 strains.

Bacteriophage Pony was isolated from a soil sample collected in College Station, TX. Phage DNA was sequenced using 454 pyrosequencing at the Emory GRA Genome Center (Emory University, Atlanta, GA). The trimmed FLX Titanium reads were assembled to a single contig at 72.9-fold coverage using the Newbler assembler version 2.5.3 (454 Life Sciences) with the default settings. The contigs were confirmed to be complete by PCR. Genes were predicted using GeneMarkS (2) and corrected using software tools available on the Center for Phage Technology (CPT) portal (https://cpt.tamu.edu/cpt-software/portal/).

Phage Pony has a 39,844-bp circularly permuted genome and 48 predicted coding sequences. It has a G+C content of 40.9% and a coding density of 96.1%. Of the 48 coding sequences, 16 were hypothetical novel, 11 were hypothetical conserved, and 21 have an annotated function. Several phage replication and recombination genes were identified. The functions assigned to those genes are molecular chaperone/plasmid replication-relaxation protein, DNA binding protein, DnaA-like replication initiator, RecF-like protein, and RecT-like recombination protein. RecF is a single-stranded DNA (ssDNA) binding protein involved in gap repair, and RecT is involved in strand invasion/recombination (3, 4). Also identified were a transcriptional regulator and an RNA polymerase sigma factor. Pony has a deoxyuridine 5′-triphosphate nucleotidohydrolase (dUTPase) involved in maintaining low cellular dUTP levels. Few structural proteins were identified by BLASTp and InterProScan (5, 6). Pony was also found to have an HNH endonuclease. A head-to-tail joining protein and a tailspike protein containing a pectin lyase domain were annotated. The pectin lyase domain has been identified as having potential antibiofilm properties (7). This can be used industrially to combat bacterial colonization of hospital environments and increasing bacterial antibiotic resistance. Pony is predicted to use a *pac* type, head-full DNA packaging mechanism as determined by TerL homology to other phages with known packaging strategies. The genes encoding phage lysis proteins include a holin gene and two possible endolysin genes. The holin is an inverted class III protein with an N-out C-in topology and a large cytoplasmic domain. The two endolysin candidates include a multidomain transglycosylase/Nlp60 peptidase protein and an l-alanyl-d-glutamate peptidase.

An interesting find is a SpoIIIE/FtsK family protein. In sporulating cells, this ATPase is generally associated with DNA translocation into the forespore (8). For the phage, this may be used in DNA injection, increasing or controlling the rate at which DNA is injected into the bacterial host cell.

### Nucleotide sequence accession number.

The genome sequence of phage Pony was contributed as accession no. KF669660 to GenBank.
